# The Triple Posterior Cruciate Ligament Sign in Bicompartmental Bucket Handle Meniscal Tear

**DOI:** 10.5334/jbsr.2472

**Published:** 2021-05-20

**Authors:** Sylvain Guillaume, Alain Bodart, Julien Collart

**Affiliations:** 1 Cliniques Saint-Pierre Ottignies, BE

**Keywords:** Bucket handle meniscal tear, anterior cruciate ligament, triple posterior cruciate ligament sign

## Abstract

**Teaching Point:** Bicompartmental bucket handle meniscal tears are very rare occurrences that can result in menisci fragments superimposition in the intercondylar notch, referred to as the triple posterior cruciate ligament sign.

## Case Study

**Figure 1:**
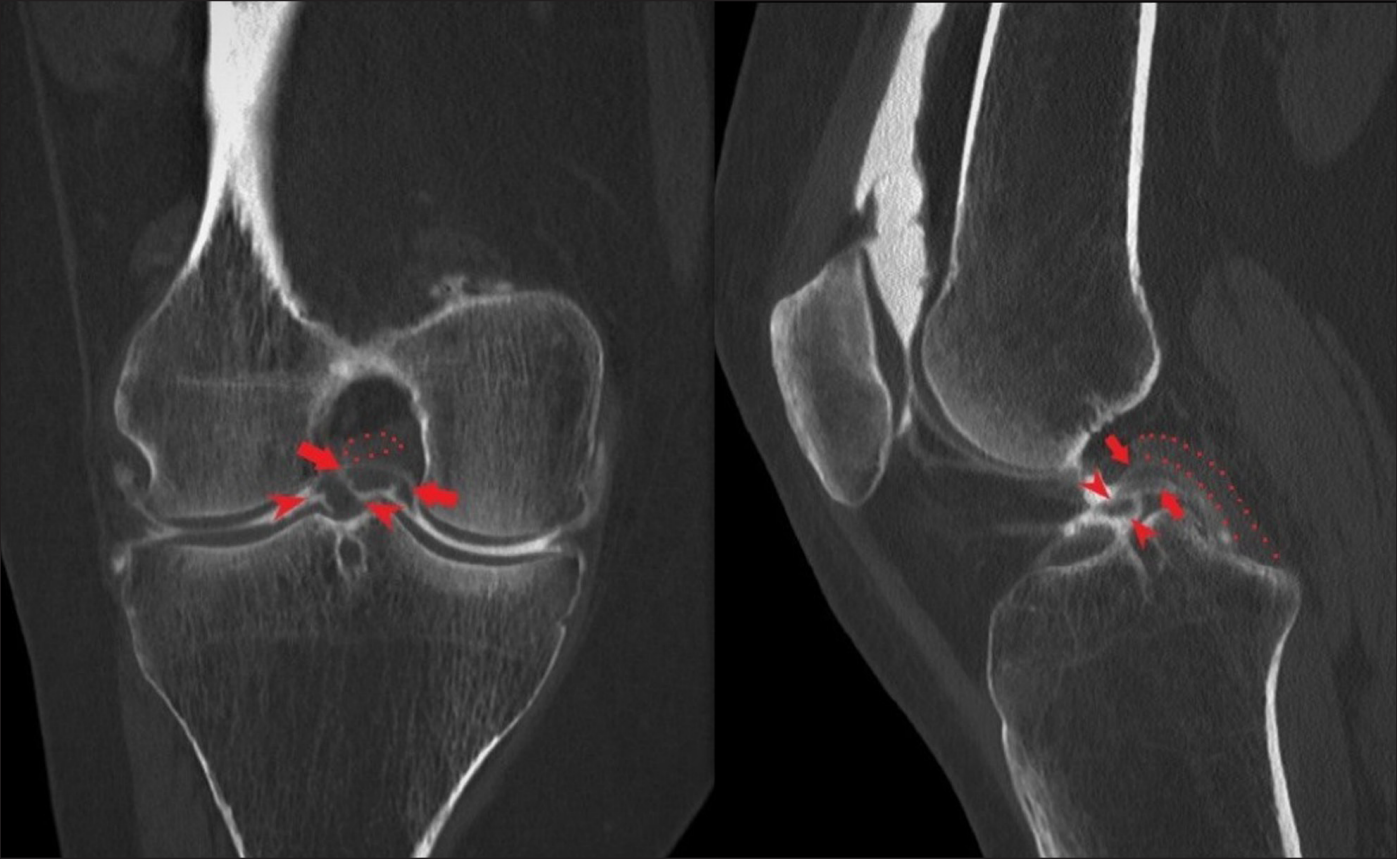


A 37-year-old woman was referred to our department to perform a knee computed tomography (CT) arthrography. She experienced pain and instability of her right knee, with a previous history of torsion trauma. The CT arthrography revealed a chronic rupture of the anterior cruciate ligament (ACL) and a bicompartmental bucket handle meniscal tear. On coronal and sagittal reformats (***Figure 1***), both lateral (arrowheads) and medial (arrows) menisci fragments were displaced in the intercondylar notch. The posterior cruciate ligament (PCL) was intact (***Figure 1***, dots). The overlap between the three structures on the sagittal reformation constitute the triple PCL sign.

## Comment

Bucket-handle meniscal tears represent approximatively 10% of all meniscal tears and occur three times more in the medial than in the lateral meniscus. Concomitant bicompartmental bucket-handle meniscal tears have rarely been reported in the literature. They mostly are the consequence of a chronic or acute lesion of the ACL [1], though exceptionally, it may be intact. The triple PCL sign is exceptional because it requires overlapping of the displaced meniscal fragments in the intercondylar notch, as in the present case. This diagnosis, which is important to manage the preoperative planning, is easily made on CT arthrography or magnetic resonance imaging.
